# Congratulations to Our Abstract Awards Winners

**DOI:** 10.1002/pmf2.12037

**Published:** 2025-01-05

**Bruce A. Work Award for Best Research by a Practicing or Training Maternal‐Fetal Medicine Physician Outside of the US**

5: Atosiban versus placebo in threatened preterm birth (APOSTEL 8‐study): an international randomized controlled trial

Larissa I. van der Windt, MD

**Disparities Award for Best Research on Diversity/Disparity in Health Outcomes**

11: Black Maternal Morbidity and its Association with Systemic Racism

Sebastian Z. Ramos, MD

**Dru Carlson Memorial Award for Best Research in Ultrasound and Genetics**

3: Fetal fraction amplification enables accurate prenatal cell‐free DNA screening at 8 weeks gestation

Lorraine Dugoff, MD

**Norman F. Gant Award for Best Research in Maternal Medicine**

58: Nifedipine versus Labetalol for Treatment of Postpartum Hypertension: A Randomized Controlled Trial

Todd R. Lovgren, MD

**The SMFM Outstanding Early Career Investigator Travel Award *Supported by Dr. Laxmi Baxi***

69: Fully Quantitative Cervical Remodeling: Race Group Differences Responsibilities and Cautions

LeAnn A. Louis, MD, MPH

